# Unclassifiable CNS tumors in DNA methylation-based classification: clinical challenges and prognostic impact

**DOI:** 10.1186/s40478-024-01728-9

**Published:** 2024-01-16

**Authors:** Richard Drexler, Florian Brembach, Jennifer Sauvigny, Franz L. Ricklefs, Alicia Eckhardt, Helena Bode, Jens Gempt, Katrin Lamszus, Manfred Westphal, Ulrich Schüller, Malte Mohme

**Affiliations:** 1 Department of Neurosurgery, University Medical Center Hamburg-Eppendorf, Martinistr. 52, 20246 Hamburg, Germany; 2 Department of Pediatric Hematology and Oncology, University Medical Center Hamburg-Eppendorf, Hamburg, Germany; 3 Research Institute Children’s Cancer Center Hamburg, University Medical Center Hamburg-Eppendorf, Hamburg, Germany; 4 Department of Radiation Hematology and Oncology, University Medical Center Hamburg-Eppendorf, Hamburg, Germany; 5 Institute of Neuropathology, University Medical Center Hamburg-Eppendorf, Hamburg, Germany

**Keywords:** Methylation, No match, Brain tumor classification, Neuropathology, CNS tumor

## Abstract

DNA methylation analysis has become a powerful tool in neuropathology. Although DNA methylation-based classification usually shows high accuracy, certain samples cannot be classified and remain clinically challenging. We aimed to gain insight into these cases from a clinical perspective. To address, central nervous system (CNS) tumors were subjected to DNA methylation profiling and classified according to their calibrated score using the DKFZ brain tumor classifier (V11.4) as “≥ 0.84” (score ≥ 0.84), “0.3–0.84” (score 0.3–0.84), or “< 0.3” (score < 0.3). Histopathology, patient characteristics, DNA input amount, and tumor purity were correlated. Clinical outcome parameters were time to treatment decision, progression-free, and overall survival. In 1481 patients, the classifier identified 69 (4.6%) tumors with an unreliable score as “< 0.3”. Younger age (*P* < 0.01) and lower tumor purity (*P* < 0.01) compromised accurate classification. A clinical impact was demonstrated as unclassifiable cases (“< 0.3”) had a longer time to treatment decision (*P* < 0.0001). In a subset of glioblastomas, these cases experienced an increased time to adjuvant treatment start (*P* < 0.001) and unfavorable survival (*P* < 0.025). Although DNA methylation profiling adds an important contribution to CNS tumor diagnostics, clinicians should be aware of a potentially longer time to treatment initiation, especially in malignant brain tumors.

## Introduction

Diagnostic profiling of central nervous system (CNS) neoplasms using genome-wide DNA methylation analysis has gained increasing importance in the field of neuropathology [1–3]. The methylation-based classification system and its diagnostic validation was initially performed by Capper et al. on 2801 CNS tumor samples and is based on a comprehensive machine learning approach [4]. The output of the classifier is a predicted probability (calibrated score) for each included CNS tumor subtype, referred to as the methylation class, which adds up to 1. As described by Capper and colleagues, the optimal trade-off between sensitivity and specificity was achieved at 0.84 [5]. Tumors with a calibrated score below 0.3 are generally classified as “no match”. Since the introduction of the method, DNA methylation profiling has become increasingly relevant and now serves as an important aid in the routine diagnostic workup of CNS tumors [5, 6]. The method facilitates a more accurate classification and differentiation of tumor subsets belonging to various entities and complements standard histopathologic examination. Recent studies have shown that the integration of a DNA methylation-based classifier resulted in a change in diagnosis in 9.8% to 25.0% of cases, which had a significant impact on the therapeutic regimen [7–9]. DNA methylation profiling is continuously advancing the field of neuro-oncology, however, there are still poorly characterized tumor types or subgroups that are difficult to define by histopathological methods as well as DNA methylation profiling. Several reports have been published on the advantages and pitfalls of DNA methylation profiling as a diagnostic tool [7–12]. These publications have noted that a proportion of 6–17% of tumors could not be assigned to a classifier diagnosis, including a significant number of pediatric or adolescent CNS tumors [7–9]. These cases pose a challenge to clinical decision making in initiating treatment as well as in selecting an optimal therapeutic regimen. To reduce the number of unclassifiable cases and improve classification specificity, an updated version of the classifier (V12.8) was recently introduced that includes a larger number of reference cases and reference groups.

To assess the clinical relevance of ambiguously classified cases, we investigated CNS tumors that were unclassifiable or had a low calibrated score using the DNA methylation-based classifier and focused on the clinical course and impact of these distinct cohorts of patients. We report our practical experience with unclear cases and compare the results between classifier version V11.4 and the updated version V12.8.

## Materials and Methods

### Study population

Data were collected from 1481 patients who underwent surgery for a CNS neoplasm and whose tumours were evaluated by genome-wide DNA methylation profiling as part of routine clinical workup between January 1, 2018, and December 31, 2021. Clinical data were collected including age, sex, type of surgery, and tumour location. Primary endpoints were time to treatment decision and number of neuro-oncology tumour boards to treatment decision. Time to treatment decision was defined as the time between surgery and final treatment recommendation by the official neuro-oncology tumour board of the University Medical Center Hamburg-Eppendorf, Hamburg, Germany.

### DNA methylation profiling

DNA was extracted from tumors and analyzed for genome-wide DNA methylation patterns using the Illumina EPIC (850 K) array. Processing of DNA methylation data was performed with custom approaches as previously described [4, 5]. The Heidelberg Brain Tumour Classifier version v11b4 was used to determine the methylation class and calibration score for each sample via www.molecularneuropathology.org [4, 5]. Patients were then divided into three groups according to their calibration score: “< 0.3” (calibration score < 0.3), “0.3–0.84” (calibration score between 0.3 and 0.84), and “≥ 0.84” (calibration score ≥ 0.84). Cut-offs for cohort separation were based on the recommendations by Capper et al. which reported a maximization of the Youden index at a calibrated score of 0.84 [4, 5]. All cases were analyzed using classifier version v11b4 and the latest version v12.8.

### DNA quality and tumor purity

The detection *p* value indicates how significantly a sample differs from the background based on its total DNA signal (unmethylated and methylated). To this end, the background is estimated by negative controls. Detection *p* values > 0.01 indicate poor DNA quality. The tumor-purity was calculated using the RF_purify Package in R [13]. This package uses the “absolute” method which measures the frequency of somatic mutations within the tumor sample and relates this to the entire DNA quantity [14].

### Statistical analysis

Differences in continuous variables were analyzed with the Mann–Whitney U test and differences in proportions were analyzed with the chi-square-test or Fisher exact test. Overall and progression-free survival was evaluated with the Kaplan–Meier method. A *p* value less than 0.05 was considered as statistically significant. All analyses were performed using SPSS Inc. Version 28 (Chicago, IL, USA). Data illustrations were performed using GraphPad Prism 10 and Adobe Illustrator 2023. Alluvial plots were graphed with R studio.

## Results

### Overview

**Fig. 1:**
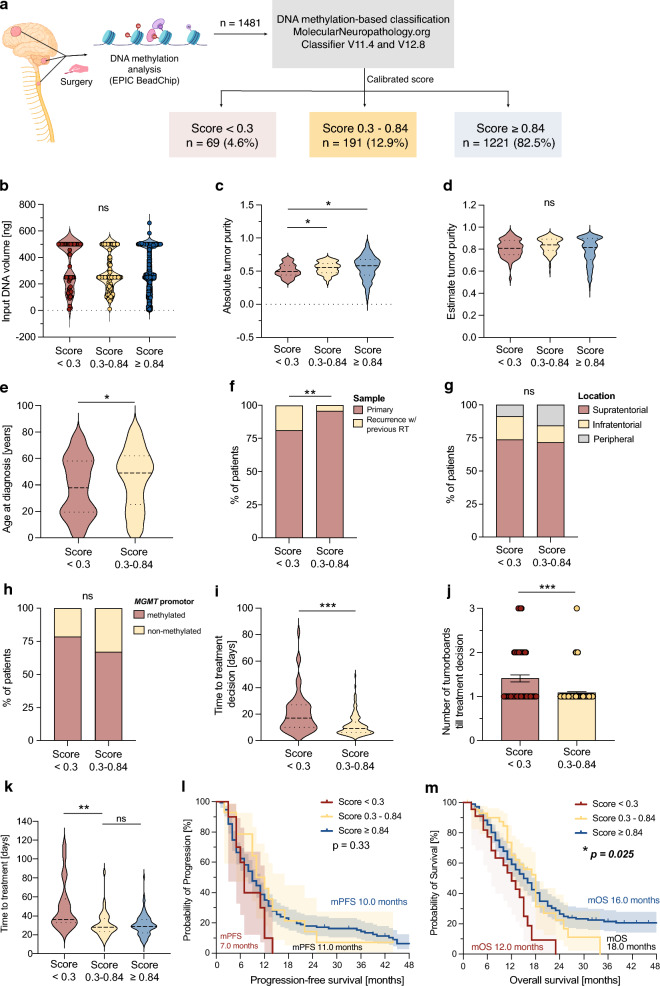
Visualization of potential confounders for the accuracy of methylation-based diagnosis. **a** Overview of the study concept. **b** DNA input amount of each sample for “< 0.3” (n = 69), “0.3–0.84” (n = 191), and “≥ 0.84” (n = 1221) cases. *ns* = *non-significant*. **c** Absolute tumor purity of each sample for “< 0.3” (n = 69), “0.3–0.84” (n = 191), and “≥ 0.84” (n = 1221) cases. *P < 0.05. **d** Relative tumor purity of each sample for “< 0.3” (n = 69), “0.3–0.84” (n = 191), and “≥ 0.84” (n = 1221) cases. ns = non-significant. **e** Age at diagnosis for “< 0.3” (n = 69) and “0.3–0.84” (n = 191) cases. *p value < 0.05. **f** Sample origin (primary tumor versus recurrent tumor after radiotherapy) for “< 0.3” (n = 69) and “0.3–0.84” (n = 191) cases. **P < 0.01. **g)** Tumor location (supratentorial versus infratentorial versus peripheral) for “< 0.3” (n = 69) and “0.3–0.84” (n = 191) cases. *ns* = *non-significant*. **h** *MGMT* methylation promotor status (non-methylated versus methylated) for “< 0.3” (n = 69) and “0.3–0.84” (n = 191) cases. *ns* = non-significant. **i** Time to treatment decision for “< 0.3” (n = 69) and “0.3–0.84” (n = 191) cases. ***P < 0.001. **j** Number of tumor boards for “< 0.3” (n = 69) and “0.3–0.84” (n = 191) cases. ***P < 0.001. **k** Time to start of adjuvant therapy in patients diagnosed with “IDH-wildtype glioblastoma” with “< 0.3”, “0.3–0.84”, and “≥ 0.84” cases. **P < 0.01, *ns* = non-significant. **l** Kaplan–Meier survival curve illustrating progression-free survival in glioblastoma cases. **m** Kaplan–Meier survival curve illustrating overall survival in glioblastoma cases

In the 4-year period studied, genome-wide DNA methylation analysis using the Illumina EPIC (850 K) array was performed on 1481 CNS tumor specimens obtained by neurosurgical resection or biopsy (Fig. 1a). Using version V11b4 of the classifier, 1221 (82.5%) samples were assigned to a methylation class with a calibration score above 0.84. Of the remaining 260 (17.6%) cases, 191 (12.9%) tumors were assigned to a methylation class with a calibration score below 0.84, while 69 (4.6%) patients could not be assigned to any methylation class and were considered as “< 0.3” (calibration score below 0.3).

### Technical aspects

Since a previous study described DNA input amount as a cause for an invalid assignment to a methylation class, we compared potential technical confounders between the three groups (Fig. 1b-d). There was no significant influence of the DNA input amount (Fig. 1b), thus even low DNA input amounts lead to a calibration score above 0.84 (range 6.8–500 ng in the match group). In contrast, the absolute tumor purity was significantly lower in the unclassifiable group “< 0.3” (Fig. 1c), but not relative tumor purity (Fig. 1d).

### Clinical aspects

Next, we examined clinical parameters for their influence on the reliability of assignment to a methylation class between the “< 0.3” group and “0.3–0.84” group. Here, we found that younger patient age at the time of material collection resulted in significantly lower chance of an accurate tumor classification (*P* = 0.03, Fig. 1e). Furthermore, there was a significantly higher percentage of samples from recurrent surgery with prior radiotherapy in the “< 0.3” group (*P* = 0.009, Fig. 1f), suggesting that radiotherapy-induced tissue remodeling compromises the diagnostic accuracy of the classifier. Location of the resected specimen (*P* = 0.26, Fig. 1g) and *MGMT* promoter methylation status (*P* = 0.13, Fig. 1h) did not influence the calibration score.

### Clinical challenges

Since DNA methylation-based classification is increasingly relevant in clinical diagnostic workflows and is considered as an extended tool in the current WHO classification [15], we sought to gain more detailed insight into the clinical and therapeutic consequences in unclassifiable cases. We defined the time until a decision for a treatment recommendation was reached and the number of required tumor boards as the primary endpoints. A comparison between the " < 0.3" and "0.3–0.84" cases revealed a significantly longer time to treatment decision (*P* < 0.0001, median 17.0 versus 9.0 days, Fig. 1i) and a higher number of tumor boards until definite treatment decision (*P* < 0.0001, Fig. 1j) in the “< 0.3” group.

In addition, we analyzed the patients with a suggested histological diagnosis of "glioblastoma, IDH-wildtype", which represented the largest subgroup among the unclassifiable cases (“< 0.3”). For further comparison, we added 180 glioblastomas cases from the " ≥ 0.84" group to this analysis. When analyzing copy number alterations, “< 0.3” glioblastoma cases showed decreased numbers of EGFR amplification (27.3% versus 39.1%, data not shown), chromosome 7 gain (31.8% versus 59.3%, data not shown), and chromosome 10 loss (31.8% versus 62.8%, data not shown) when compared to the “≥ 0.84” group.

Patients assigned to the “< 0.3” group had a significantly longer time to initiation of adjuvant therapy (*P* < 0.001, median 38.5 days versus 28.0 days versus 29.0 days, Fig. 1k). While the progression-free survival did not differ significantly between the groups (*P* = 0.33, median 7.0 months versus 11.0 months versus 10.0 months, Fig. 1l), patients with “< 0.3” tumors displayed a significantly shorter overall survival (*P* = 0.025, median 12.0 months versus 18.0 months versus 16.0 months, Fig. 1m). Collectively, these findings indicate that difficulties assigning a diagnosis accurately through methylation profiling has a significant adverse clinical impact.

### Histopathology and change of diagnoses using v12.8

**Fig. 2:**
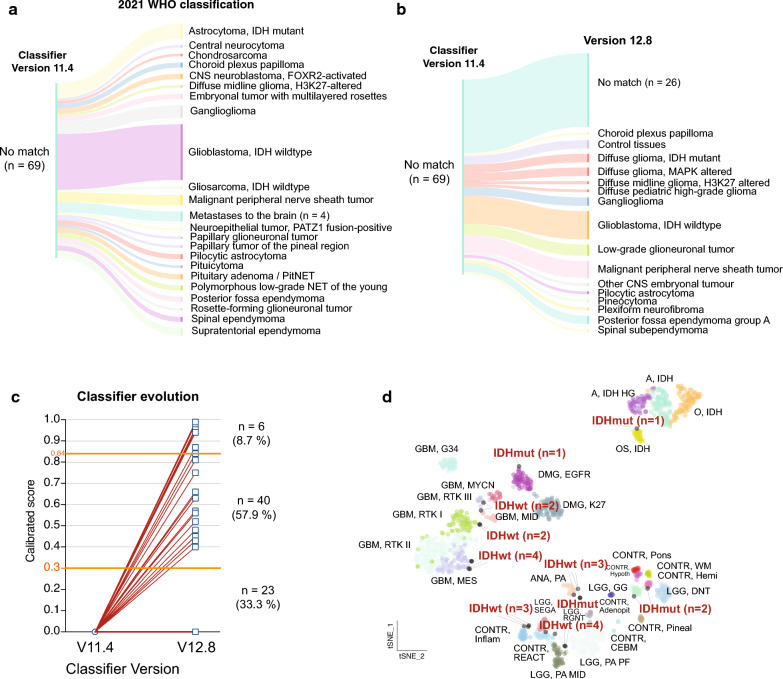
Histopathological and methylation-based diagnoses in the “no match” (“< 0.3”) cohort. **a** Sankey plot illustrating the histological diagnoses of unclassifiable cases using the 2021 WHO classification. **b** Sankey plot illustrating the new assigned methylation class using the latest classifier version. **c** Change of the calibrated score using the latest classifier version for all unclassifiable cases. All cases using classifier version 11.4 are centered at zero, as no specific calibration score is available in this version. **d** t-Distributed Stochastic Neighbor Embedding (tSNE) of non-matching glioma samples and matching gliomas and control tissue from the Capper et al. reference data set [4]. Non-matching IDHwt and IDHmut samples are highlighted in red

We further listed histopathological diagnosis based on the current WHO classification [15] and classifier output. The most common histological diagnoses were "glioblastoma, IDH-wildtype" (n = 22, 31.9%) "astrocytoma, IDH-mutant" (n = 6, 8.7%), “ganglioglioma” (n = 6, 8.7%), and “malignant peripheral nerve sheath tumor” (n = 5, 7.2%) (Fig. 2a). Of samples diagnosed as “glioblastoma, IDH-wildtype”, 5 of the 22 (22.7%) cases were obtained from recurrent surgery after radio-chemotherapy, indicating that treatment can affect the diagnostic accuracy of methylation-based classification.

The classifier algorithm is continuously being improved [5, 6] and new versions such as v12.8 have been added since its original release. Reclassification of the “< 0.3” cases by classifier version 12.8 was able to assign a diagnosis in 46 of 69 (66.7%) cases (Fig. 2b) with 6 cases achieving a calibrated score above 0.84 (Fig. 2c). However, 23 (33.3%) cases remained unclassifiable (Fig. 2b-c). The samples with a newly assigned diagnosis in the updated classifier version were most frequently diagnosed as "glioblastoma, IDH-wildtype" (n = 10), “malignant peripheral nerve sheath tumor” (n = 5), ependymoma groups (n = 4) and “ganglioglioma” (n = 3) (Fig. 2b).

Unclassifiable glioma samples (n = 23) were additionally plotted in a t-distributed stochastic neighbor embedding (t-SNE) of glioma and control tissue. The t-SNE plot assigns the sample location according to similarities of epigenetic profiles, thereby enabling an approximation of the diagnosis beyond the matching score alone. Figure 2d shows that both, non-classifiable IDHwt and IDHmut glioma samples can be approximated to defined molecular subgroups. Although visual analysis of the tSNE plot does not provide a quantifiable output, it is obvious that only five IDHwt cases primarily grouped with control tissue samples (Fig. 2d), whereas all the other non-classifiable epigenetic profiles could be approximated to a defined molecular tumor subtype. Such approximation could potentially guide further molecular diagnostic workup in ambiguous cases.

## Discussion

The decision about appropriate treatment options for patients with CNS tumors depends on reliable and accurate diagnosis. Given the molecular heterogeneity of CNS neoplasms and the increasing numbers of distinct tumor subgroups varying in clinical course, precise diagnostic classification can be difficult and poses a major challenge for neuropathologists and neurooncologists. In recent years, DNA methylation-based tumor classification has emerged as an additional and powerful tool, extending routine diagnostics and is becoming increasingly important in the neuro-oncology field [4–6, 12, 16]. In 2018, Capper and colleagues described the Heidelberg experience and shared their recommendations and approaches, which represented a fundamental step toward implementing DNA methylation-based classification into clinical practice [5]. However, cases exist that are unclassifiable and cannot be assigned to any methylation class, which is challenging for finding an optimal therapy. Our study focused on the clinical course in these difficult cases and presents the following findings: (1) 4.6% of cases were not accurately classified with a calibrated score below 0.3 and an additional 12.9% had a low calibrated score among the CNS tumor samples examined at our institution. (2) Lower absolute tumor purity, younger age, and recurrent tumor tissue post radiotherapy hindered accurate classification. (3) " < 0.3" cases had a significantly higher number of tumor board presentations and longer time to treatment decision than matched cases. (4) In the subset of IDH wildtype glioblastomas, unclassifiable cases were found to have significantly longer time to initiation of adjuvant treatment and less favorable overall survival.

A total of 1481 cases submitted to genome-wide DNA methylation profiling using the Illumina EPIC (850 K) array as part of the diagnostic workup were included in our study. First, we investigated technical as well as clinical factors influencing the calibrated score. Wu et al. reported the precision of the Heidelberg classifier in 1258 cases and identified a DNA input lower than 100 ng as well as low tumor purity as confounding factors related to the calibrated score [16]. While we detected no correlation with DNA input amount in our study, absolute DNA tumor purity was also shown to be a detrimental factor for the accuracy of the classifier. From the clinical perspective, younger age and samples obtained from tumor recurrences were unfavorable factors for diagnostic accuracy. Regarding the recurrent samples, most patients had undergone prior radiotherapy, suggesting post radiogenic tissue remodeling that impedes classification. The lower accuracy at younger patient age was recently addressed by Capper and colleagues and seems likely to improve in the future [6]. Additionally, application of further methods such as next generation sequencing might be helpful for a more precise diagnosis in challenging cases since a recent study showed a correlation between variant allelic frequency, sample cellularity, and DNA methylation profiling success [17].

Previous studies have shown that integration of the DNA methylation-based classifier resulted in a change of diagnosis in 9.8% to 25.0% of cases, which had a significant impact on the treatment regimen [7–9]. Karimi et al. presented seven cases in which methylation profiling directly impacted patient care, avoiding potentially inadequate treatment [10]. However, in malignant CNS tumors such as high-grade gliomas which require adjuvant radio- and/or chemotherapy, a delay in treatment initiation due to continued diagnostic investigation could potentially impact patient outcome [18, 19]. Although the optimal timing for treatment initiation is widely debated, evidence suggests that a treatment start later than eight weeks after surgical resection could lead to poorer survival in high-grade gliomas [18–21]. With this in mind, we observed a significantly longer time to final treatment decision in unclassifiable cases. To further investigate this aspect, we analyzed a subset with the histologic diagnosis of IDH-wildtype glioblastoma. Here, a significantly longer time interval between surgery and initiation of adjuvant therapy was also observed in unclassifiable cases. This was further reflected in shorter overall survival in this distinct cohort of patients. Therefore, we recommend that adjuvant treatment should be planned as early as possible when surgical and histological results are suspicious of IDH-wildtype glioblastoma, as an unfavorable clinical impact has been demonstrated in these challenging cases.

A major advantage is offered by the constant advancement with updated versions. We reclassified our cases with the latest version, where 62.3% of “< 0.3” cases could be assigned a methylation class. Even though most difficult cases could now be assigned to a matching methylation class, it is worth noting that some cases were still unclassifiable. In these patients, it may be critical to find the optimal therapeutic regimen, and time to treatment initiation should be considered. Previously published recommendations to increase accuracy by using deconvolution as an additional tool are promising approaches for unclassifiable cases, but seem difficult to apply in daily clinical routine [16].

In the future, the constant improvement of classifier accuracy by the enlargement of reference cohorts and adaptation of algorithms as well as the incorporation of additional bioinformatic tools in the diagnostic workup can be expected to further increase the accuracy of CNS tumor classification.

## Conclusion

Our study demonstrates the clinical challenges in CNS tumors unclassifiable by methylation profiling and highlights the impact on treatment delay when waiting for an accurate diagnosis. Although DNA methylation profiling adds an important contribution to advanced CNS tumor diagnosis, clinicians should be aware of a potentially longer time to treatment initiation, especially in highly malignant brain tumors.

## Data Availability

All data and idat files are available from the corresponding author upon reasonable request.

## References

[1] Khalsa SSS, Hollon TC, Adapa A (2020). Automated histologic diagnosis of CNS tumors with machine learning. CNS Oncol.

[2] Rauschert S, Raubenheimer K, Melton PE, Huang RC (2020). Machine learning and clinical epigenetics: a review of challenges for diagnosis and classification. Clin Epigenet.

[3] Pickles JC, Stone TJ, Jacques TS (2020). Methylation-based algorithms for diagnosis: experience from neuro-oncology. J Pathol.

[4] Capper D, Jones DTW, Sill M (2018). DNA methylation-based classification of central nervous system tumours. Nature.

[5] Capper D, Stichel D, Sahm F (2018). Practical implementation of DNA methylation and copy-number-based CNS tumor diagnostics: the Heidelberg experience. Acta Neuropathol (Berl).

[6] Sturm D, Capper D, Andreiuolo F (2023). Multiomic neuropathology improves diagnostic accuracy in pediatric neuro-oncology. Nat Med.

[7] Pages M, Uro-Coste E, Colin C (2021). The implementation of DNA methylation profiling into a multistep diagnostic process in pediatric neuropathology: a 2-year real-world experience by the French neuropathology network. Cancers.

[8] Priesterbach-Ackley LP, Boldt HB, Petersen JK (2020). Brain tumour diagnostics using a DNA methylation-based classifier as a diagnostic support tool. Neuropathol Appl Neurobiol.

[9] Jaunmuktane Z, Capper D, Jones DTW (2019). Methylation array profiling of adult brain tumours: diagnostic outcomes in a large, single centre. Acta Neuropathol Commun.

[10] Karimi S, Zuccato JA, Mamatjan Y (2019). The central nervous system tumor methylation classifier changes neuro-oncology practice for challenging brain tumor diagnoses and directly impacts patient care. Clin Epigenet.

[11] Ferreyra Vega S, Olsson Bontell T, Corell A, Smits A, Jakola AS, Carén H (2021). DNA methylation profiling for molecular classification of adult diffuse lower-grade gliomas. Clin Epigenet.

[12] Pickles JC, Fairchild AR, Stone TJ (2020). DNA methylation-based profiling for paediatric CNS tumour diagnosis and treatment: a population-based study. Lancet Child Adolesc Health.

[13] Johann PD, Jäger N, Pfister SM, Sill M (2019). RF_Purify: a novel tool for comprehensive analysis of tumor-purity in methylation array data based on random forest regression. BMC Bioinform.

[14] Carter SL, Cibulskis K, Helman E (2012). Absolute quantification of somatic DNA alterations in human cancer. Nat Biotechnol.

[15] Louis DN, Perry A, Wesseling P (2021). The 2021 WHO classification of tumors of the central nervous system: a summary. Neuro-Oncol.

[16] Wu Z, Abdullaev Z, Pratt D (2021). Impact of the methylation classifier and ancillary methods on CNS tumor diagnostics. Neuro Oncol.

[17] Jamshidi P, McCord M, Galbraith K (2023). Variant allelic frequency of driver mutations predicts success of genomic DNA methylation classification in central nervous system tumors. Acta Neuropathol (Berl).

[18] Irwin C, Hunn M, Purdie G, Hamilton D (2007). Delay in radiotherapy shortens survival in patients with high grade glioma. J Neurooncol.

[19] Gliński B, Urbański J, Hetnał M (2012). Prognostic value of the interval from surgery to initiation of radiation therapy in correlation with some histo-clinical parameters in patients with malignant supratentorial gliomas. Współczesna Onkol.

[20] Buszek SM, Al Feghali KA, Elhalawani H, Chevli N, Allen PK, Chung C (2020). Optimal timing of radiotherapy following gross total or subtotal resection of glioblastoma: a real-world assessment using the national cancer database. Sci Rep.

[21] Blumenthal DT, Won M, Mehta MP (2009). Short delay in initiation of radiotherapy may not affect outcome of patients with glioblastoma: a secondary analysis from the radiation therapy oncology group database. J Clin Oncol.

